# Electrocochleography for Monitoring Hearing Preservation During Cochlear Implantation

**DOI:** 10.1001/jamaoto.2025.4044

**Published:** 2025-11-20

**Authors:** Raphael R. Andonie, Christofer Bester, Sudanthi Wijewickrema, Leanne Sijgers, Marlies Geys, Adrian Dalbert, Marco Caversaccio, Flurin Pfiffner, Stephen O’Leary, Stefan Weder

**Affiliations:** 1 Department of Otorhinolaryngology–Head & Neck Surgery, Bern University Hospital, Bern, Switzerland; 2 ARTORG Center for Biomedical Engineering Research, University of Bern, Bern, Switzerland; 3 Departments of Surgery and Otolaryngology, University of Melbourne, Melbourne, Australia; 4 Royal Victorian Eye and Ear Hospital, Melbourne, Australia; 5 Department of Otorhinolaryngology–Head & Neck Surgery, University Hospital Zurich, University of Zurich, Zurich, Switzerland

## Abstract

**Importance:**

Many cochlear implant candidates retain residual hearing, which can improve speech perception and overall outcomes. However, residual hearing is frequently lost during implantation. Intraoperative electrocochleography may enable monitoring of residual hearing, but its clinical relevance is currently limited due to reliance on expert interpretation, the absence of real-time analysis algorithms, and signal variability.

**Objective:**

To assess whether automated analysis of intraoperative cochlear microphonic amplitude decreases (events) is associated with hearing preservation and to evaluate additional electrocochleography features enhancing potential predictive performance.

**Design, Setting, and Participants:**

This multicenter, cross-sectional cohort study included adults receiving a cochlear implant with a preoperative hearing threshold not greater than 85 dB hearing level at 0.5 kHz and cochlear microphonic amplitude of 5 μV or higher across 3 tertiary referral centers in Melbourne, Australia; Bern, Switzerland; and Zurich, Switzerland. Intraoperative real-time electrocochleography and impedance data were collected between 2017 and 2025, with audiometric follow-up 3 months postoperatively.

**Exposures:**

Cochlear implantation with the Slim Straight Electrode array (Cochlear Limited).

**Main Outcomes and Measures:**

Primary outcome was binary hearing preservation at 3 months postoperatively, defined as less than 25% loss relative to the preoperative low-frequency pure-tone average at 0.25 to 1 kHz. Primary variables assessed as possible predictors included magnitude, location, and persistence of cochlear microphonic events. Post hoc analysis explored associated changes in the auditory nerve neurophonic:cochlear microphonic amplitude ratio and cochlear microphonic phase.

**Results:**

A total of 112 patients met inclusion criteria and were analyzed (median [IQR] age at surgery, 68 [58-75] years; 57 [51%] female and 55 [49%] female). Cochlear microphonic events persisting or occurring near the end of insertion were associated with loss of residual hearing (adjusted odds ratio, 31.58 [95% CI, 6.36-205.36] and 52.96 [95% CI, 8.02-472.63], respectively), independent of age, preoperative hearing, and participating hospital. Events with rising amplitude ratio between auditory nerve neurophonic and cochlear microphonic were associated with better hearing preservation (mean difference in hearing preservation, 24.4% [95% CI, 7.3%-41.5%]). Stable cochlear microphonic phase showed a similar trend (mean difference in hearing preservation, 20.9% [95% CI, 2.9%-38.9%]).

**Conclusions and Relevance:**

This cross-sectional study demonstrates the feasibility of automated intraoperative electrocochleography for possible prediction of hearing preservation during cochlear implantation. Persistent cochlear microphonic events near the end of insertion were associated with hearing loss. Additional electrocochleography features may improve signal interpretation. These findings support the development of real-time feedback systems to guide cochlear implantation.

## Key Points

**Question:**

Is automated intraoperative monitoring of residual hearing during cochlear implantation feasible using objective electrocochleography analysis?

**Findings:**

In this cross-sectional study of 112 adults receiving cochlear implants, decreases in cochlear microphonic amplitude were associated with postoperative hearing loss when they were persistent or occurred near the end of electrode insertion. Hearing preservation was particularly poor when the amplitude ratio between the auditory nerve neurophonic and cochlear microphonic did not increase or when the cochlear microphonic phase remained stable.

**Meaning:**

Automatically extracted electrocochleography features may predict residual hearing outcomes during cochlear implantation, and these findings support the development of real-time feedback systems assisting surgeons during implantation.

## Teaser

This cross-sectional study assesses whether automated analysis of intraoperative cochlear microphonic amplitude decreases is associated with hearing preservation.

## Introduction

Cochlear implantation provides effective auditory rehabilitation for individuals with moderate to profound sensorineural hearing loss.^1,2,3^ Currently, many cochlear implant candidates present with residual low-frequency hearing, which enhances speech perception if preserved despite implantation.^4,5^ However, half of these patients lose residual hearing during or after surgery.^3^ Poor hearing preservation outcomes have been linked to mechanical blocking of sound transmission by the implant or surgical trauma.^6,7,8^

To improve hearing preservation, intraoperative functional monitoring via electrocochleography is being actively researched.^9,10^ This technique typically uses the apical cochlear implant electrode to record acoustically evoked potentials of the sensory and nerve cells in the inner ear, including the cochlear microphonic (CM) and the auditory nerve neurophonic (ANN).^11^ Mostly, the CM amplitude is isolated from the composite electrocochleography responses and analyzed, as it usually exhibits the largest amplitude.^10^ Multiple studies have reported that CM events (ie, decreases in CM amplitude above a preset detection threshold, such as a drop of 30% from the prior maximum) indicate poor hearing preservation.^10,12,13,14^ Furthermore, surgical interventions, such as pausing the insertion and repositioning the electrode in response to CM events, have been shown to improve hearing preservation.^15^

Yet, interpreting CM events remains controversial, as signal fluctuations have been observed even when hearing was preserved. Said fluctuations are hypothesized to result from interference between hair cell and neural potentials—constructive or destructive depending on their relative strength and phase—or from the measurement electrode passing over discrete clusters of residual hair cells.^16,17,18,19^ Since both phenomena can occur independently of surgery-related trauma, CM amplitude drops in the real-time recordings do not necessarily indicate a loss of residual hearing.^16,20,21^ As a result, CM-based prediction of hearing preservation shows low sensitivity and specificity.^9^ Recent efforts to improve prediction have focused on complementing CM amplitude by analyzing the ANN:CM ratio or CM phase, with encouraging results.^16,20,22,23,24^

Nevertheless, the clinical relevance of electrocochleography for monitoring residual hearing remains minimal.^25^ This is largely due to the absence of software capable of automatically analyzing electrocochleography signals and providing clear, intuitive feedback to the surgeon.^26^ In addition, many studies rely on subjective expert interpretation and prior assumptions, which limits the reproducibility of their findings.^15,22^

To address these limitations, this multicenter, cross-sectional cohort study implements an objective electrocochleography analysis pipeline for automatically assessing individual hearing preservation during cochlear implantation. We hypothesize that automated processing of real-time electrocochleography recordings is technically feasible and may help predict hearing preservation outcomes through multimodal signal analysis. Building on this, we evaluated CM events using logistic regression to assess their possible predictive value for hearing preservation, applying different paradigms based on location and persistence. A secondary analysis evaluated whether the ANN:CM ratio and CM phase can be used to enhance the interpretation of CM events.

## Methods

### Study Design

Eligible were adults receiving a cochlear implant with the Slim Straight Electrode array (Cochlear Limited) and a preoperative hearing threshold not greater than 85 dB hearing level (dB HL) at 0.5 kHz. All implantations used a posterior tympanotomy approach, followed by manual electrode array insertion through the round window. Surgeons adhered to common soft surgery techniques. Patients were included in the analysis if CM amplitudes exceeded 5 μV for at least 10 seconds during electrode insertion.

This study adhered to the Declaration of Helsinki and received approval from local institutional review boards in Melbourne, Australia; Bern, Switzerland; and Zurich, Switzerland. Participants provided written informed consent before study enrollment.

### Audiometry

Unaided pure-tone air-conduction thresholds in dB HL were recorded at frequencies of 0.25, 0.5, and 1.0 kHz before surgery and 3 months thereafter. Following Skarzynski et al,^27^ the pure-tone average (PTA) was calculated from these frequencies to derive relative hearing preservation (HP). In this scale, 0% reflects complete loss of residual hearing (ie, the dynamic range between preoperative PTA and the maximum measurable PTA of the used audiometer, which was 115 dB HL), while 100% indicates full preservation. Binary hearing preservation outcomes were defined as hearing preserved if HP was 75% or higher and hearing lost if HP was less than 75%.^27^

### Intraoperative Electrocochleography

During electrode insertion, observational electrocochleography (ie, no feedback to the surgeon and no electrocochleography-based intervention) was continuously recorded at the most apical cochlear implant electrode. Measurements were performed using the cochlear implant’s telemetry system and controlled via a research laptop using the University of Melbourne’s Cochlear Response Telemetry investigational software, developed in collaboration with Cochlear Limited, or its commercial derivatives (Cochlear Research Platform, version 2.0 [Cochlear Limited]). Acoustic stimuli (0.5 kHz tone bursts lasting 6-13 milliseconds at 100-110 dB HL to elicit large electrocochleography responses) were delivered through foam insertion ear tips positioned in the external auditory canal and connected to a calibrated acoustic output device via a 25-mm plastic sound tube. Condensation and rarefaction responses were recorded every 700 milliseconds, with a sampling rate of 20 kHz.^14,28,29^ CM amplitude, CM phase, and ANN amplitude were automatically extracted from each condensation-rarefaction pair using the algorithms described in Andonie et al^30^ and Bester et al^20^ in an observational window of 200 seconds. The electrocochleography signals were denoised using a backward-moving average filter with a window length of 4.7 seconds before further analysis.

Simultaneously, we recorded electrode impedance to automatically detect entry of the basal electrode into the cochlea by an impedance drop of more than 50%.^13^ For reference, we estimated the angular insertion depth at this stage to be roughly 330°, applying the formula provided by Anschuetz et al,^31^ with an average cochlear diameter of 9.23 mm and 19.1-mm distance of the basal electrode from the tip of the electrode array.^32,33^ When no impedance measurements were available (12 cases), we used insertion tags based on live surgeon commentary.

### Automated Extraction of CM Events

CM events were extracted from electrocochleography recordings using a negative edge filter, as described in Wildhaber et al^34^ and Waldmann et al.^35^ This algorithm detects negative signal edges by recursively evaluating a finite difference operator (similar to a derivative kernel or 1-dimentional Sobel filter) in a 5-second sliding window. The amplitude drop was calculated with respect to the amplitude at the start and at the end of each edge.

To assess the influence of CM event location and persistence on hearing preservation prediction in the subsequent analysis, 4 evaluation paradigms were defined: (1) naive, including all events; (2) deep, including only events occurring after insertion of the basal electrode; (3) persistent, including only events after which the CM amplitude did not recover; and (4) combined, merging the latter 2. Per patient, only the CM event with the largest amplitude drop was considered.

### Statistical Analysis

We used logistic regression to evaluate the potential predictive value of intraoperative CM events for hearing preservation outcomes as the binary dependent variable. The primary independent variable was the amplitude drop associated to a CM event. Both univariate and multivariable models were used, with the latter controlling for preoperative PTA, age, and implantation center. We did not adjust for sex or side, as previous studies have consistently shown no meaningful effect on hearing preservation outcomes.^36,37^ In 3 cases where the exact age at the time of surgery was missing, we applied mean imputation. To quantify the estimated effect size of each possible predictor, we calculated the corresponding odds ratio (OR) and 95% CI. For each of the 4 CM event paradigms (naive, deep, persistent, and combined), we fitted separate logistic regression models to evaluate their respective predictive value. The statistical analysis was implemented using R, version 4.3.1 (R Project for Statistical Computing).

### Explorative Post Hoc Analysis of Additional Electrocochleography Features

**Figure 1.**
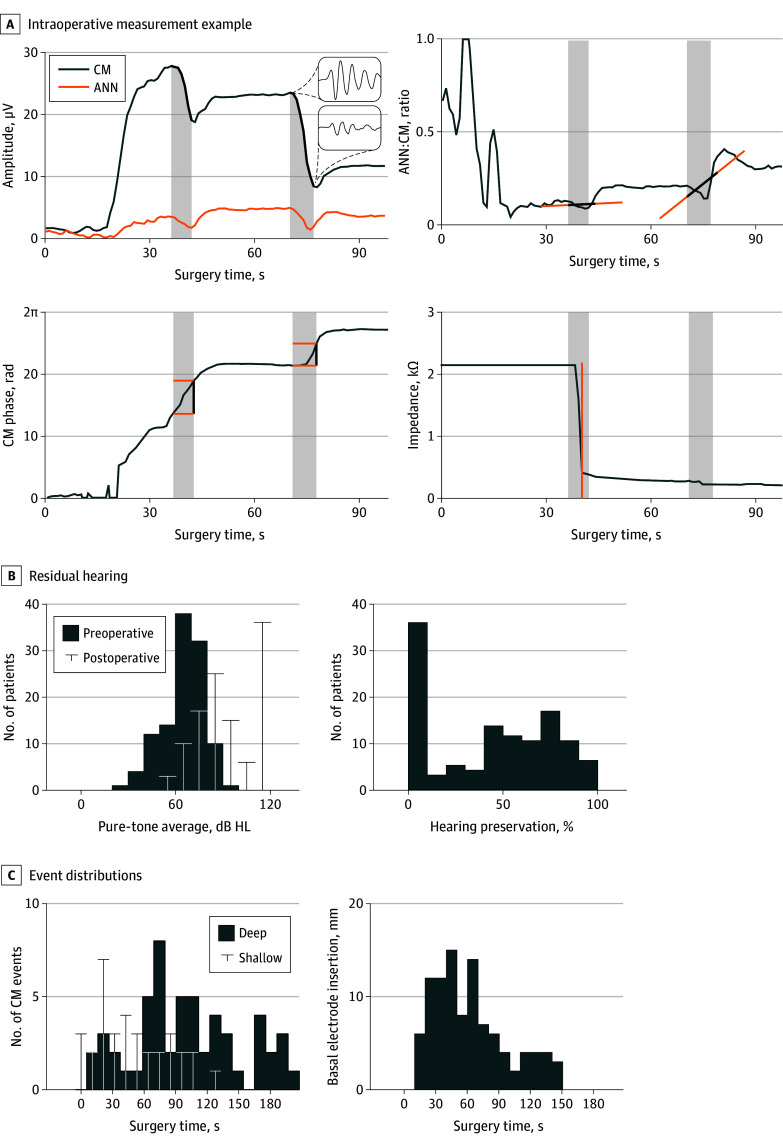
Intraoperative Electrocochleography Measurement and Population Data Overview A, Example of real-time electrocochleography recording during cochlear implant electrode insertion. The top panel shows the evolution of cochlear microphonic (CM) and auditory nerve neurophonic (ANN) amplitudes, with CM amplitude drops (CM events) highlighted. Insets show single responses before and after the major CM event. The second and third panels illustrate the ANN:CM amplitude ratio and CM phase evolution, respectively, with the additionally extracted event features. The bottom panel displays electrode impedance changes, with the orange line marking the estimated cochlear entry time of the basal electrode. Shaded areas indicate the temporal domain of the drop. B, Population-level residual hearing outcomes showing distributions of preoperative and postoperative pure-tone averages in decibel hearing level (dB HL) (left) and relative hearing preservation (right). C, Distribution of measurement events over insertion time. The left panel shows histograms of CM events extracted using an amplitude cutoff of 0.36, stratified by intracochlear location of occurrence (ie, before [shallow] or after [deep] insertion of the basal-most electrode). The right panel shows the histogram of estimated times at which the basal electrode was inserted into the cochlea. Rad indicates radian.

In a post hoc analysis, we explored the potential of the ANN:CM ratio and CM phase for enhancing the interpretation of CM events above the cutoffs identified in the univariate models. Thereby, we compared hearing preservation of patients showing CM events with increasing ANN:CM ratio and stable CM phase to those without. For reference, we also compared patients with CM events above the cutoffs to those without. The ANN:CM ratio was analyzed only when it exceeded 0.25, and CM phase was evaluated only when CM amplitude was at least 10 μV. ANN:CM ratio increases were identified following Bester et al,^20^ evaluating the signal from the start to 2 seconds after each CM event. Automating the approach from Dalbert et al,^22^ CM phase shifts were detected when exceeding a 10% range (36°) during the event. The feature extraction is visualized in Figure 1A.

We reported the difference in group means and the corresponding 95% CIs, calculated using t-statistic for 2 independent samples without assuming equal variances.^38^ The post hoc analysis was conducted using Python, version 3.10.12, with the SciPy, version 1.11.1, package (Python Software Foundation).^39,40^

## Results

### Participants and Data Overview

Of the 145 patients, 112 (77%) showed CM amplitudes exceeding 5 μV and were included in the analysis, with 78 from Melbourne, 22 from Bern, and 12 from Zurich. Of these patients, 57 (51%) identified as female and 55 (49%) as male, with a median (IQR) age at surgery of 68 (58-75) years.

Preoperative PTAs (median [IQR], 66 [57-73] dB HL) deteriorated postoperatively (median [IQR], 90 [77-115] dB HL), except in 4 patients, where the PTA remained unchanged. Relative hearing preservation was distributed bimodally (median [IQR], 48% [0%-72%]), as shown in Figure 1B. Hearing was preserved (binary outcome) in 26 patients (23% of the study population), while being lost in 86 patients (77% of the study population).

Electrocochleography recordings varied across patients and over time (median [IQR] CM amplitude, 10.16 [4.40-19.39] μV; median [IQR] ANN amplitude, 2.05 [1.13-3.69] μV). CM events were most frequent after insertion of the basal-most electrode (median [IQR] entry time, 56 [37-82] seconds) (Figure 1C). Applying the different paradigms to the 112 patients in whom CM events were detected resulted in 88 patients evaluated as naive, 62 as deep, 62 as persistent, and 71 as combined.

### Association of CM Events and Hearing Preservation

**Figure 2.**
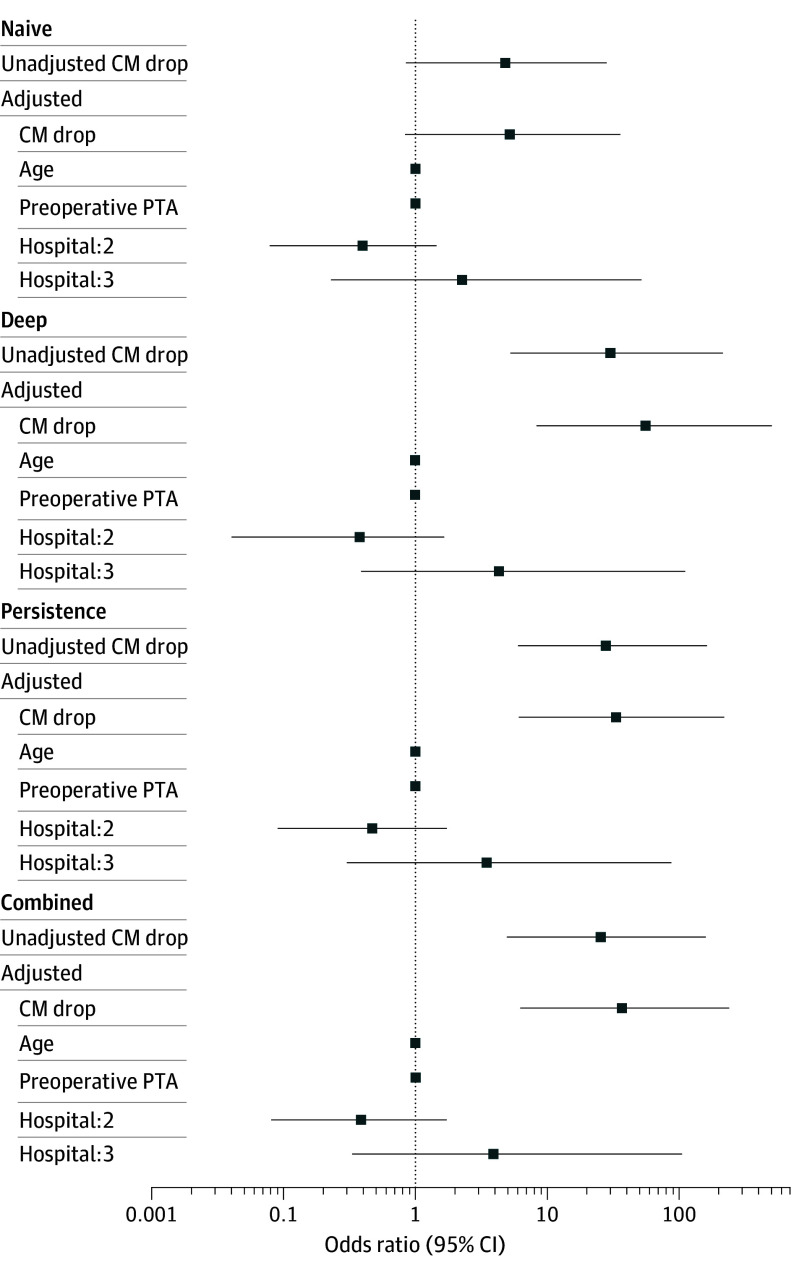
Forest Plot from Univariate and Multivariable Logistic Regression Models Across Cochlear Microphonic (CM) Event Paradigms Each row represents 1 possible predictor, plotted on a logarithmic odds ratio scale. Horizontal bars indicate 95% CIs, and the vertical dotted line marks an odds ratio of 1 (no effect estimate). All numeric values presented in this graph are also provided in Table 1.

Both univariate and multivariable logistic regression similarly indicated CM events as associated with binary hearing preservation outcomes. Estimated effect size and model discrimination varied across the analyzed sets of CM events, suggesting persistent events near the end of electrode insertion as the strongest possible predictors (deep: adjusted OR, 52.96 [95% CI, 8.02-472.63]; persistent: adjusted OR, 31.58 [95% CI, 6.36-205.36]; combined: adjusted OR, 35.24 [95% CI, 6.03-262.23]; and naive: adjusted OR, 5.05 [95% CI, 0.82-33.22]). Although CM events showed higher effect estimates for hearing preservation, the wide upper confidence limits reflect uncertainty likely due to class imbalance. Potential confounders, including preoperative PTA, age, and implantation center, did not contribute meaningfully. These results are summarized in Table 1 and depicted in Figure 2.

**Table 1.** Logistic Regression Results for the Association of Binary Hearing Preservation Outcomes With Cochlear Microphonic (CM) Evaluation Paradigms

Paradigm	Variable^a^	No. of patients with CM events^b^	Odds ratio (95% CI)^c^
Naive, unadjusted	Intercept	88	0.78 (0.27-2.12)
	CM drop		4.60 (0.84-26.63)
Naive, adjusted	Intercept		1.44 (0.04-59.01)
	CM drop		5.05 (0.82-33.22)
	Preoperative PTA		0.99 (0.95-1.03)
	Age		1.02 (0.99-1.05)
	Hospital:2^d^		0.39 (0.08-1.38)
	Hospital:3^d^		2.17 (0.23-49.13)
Deep, unadjusted	Intercept	62	1.24 (0.58-2.67)
	CM drop		28.50 (5.17-194.70)
Deep, adjusted	Intercept		0.62 (0.01-30.80)
	CM drop		52.96 (8.02-472.63)
	Preoperative PTA		0.99 (0.95-1.03)
	Age		1.02 (0.99-1.06)
	Hospital:2^d^		0.37 (0.07-1.45)
	Hospital:3^d^		4.19 (0.38-102.74)
Persistent, unadjusted	Intercept	62	1.06 (0.56-2.02)
	CM drop		26.79 (5.89-153.73)
Persistent, adjusted	Intercept		0.62 (0.01-36.97)
	CM drop		31.58 (6.36-205.36)
	Preoperative PTA		0.99 (0.95-1.03)
	Age		1.02 (0.99-1.06)
	Hospital:2^d^		0.46 (0.09-1.83)
	Hospital:3^d^		3.34 (0.30-83.52)
Combined, unadjusted	Intercept	71	1.42 (0.62-3.29)
	CM drop		24.49 (4.79-150.19)
Combined, adjusted	Intercept		0.37 (0.01-20.00)
	CM drop		35.24 (6.03-262.23)
	Preoperative PTA		0.99 (0.95-1.03)
	Age		1.03 (0.99-1.06)
	Hospital:2^d^		0.43 (0.08-1.66)
	Hospital:3^d^		3.76 (0.33-96.68)

Abbreviation: PTA, pure-tone average.

^a^ Variables assessed as possible predictors.

^b^ Out of a total of 112 patients.

^c^ Odds ratio indicates lost-to-preserved hearing.

^d^ Values indicate participating implantation centers randomly enumerated.

### Post Hoc Exploration of Additional Electrocochleography Features

Group separation using CM event cutoffs derived from the univariate models (relative amplitude change, 36.4%-39.4%) were different across evaluation paradigms. The mean difference in HP across the different paradigms was 19.2% (95% CI, 4.4%-33.9%) for naive, 18.3% (95% CI, 5.9%-30.7%) for deep, 26.2% (95% CI, 14.3%-38.0%) for persistent, and 24.2% (95% CI, 11.7%-36.7%) for combined.

The ANN:CM ratio was sufficient for analysis in 67.7% to 79.5% of the CM events, in which it increased in 75.7% to 82.6%. Patients with increasing ANN:CM ratio showed higher hearing preservation (mean difference in HP: naive: 12.2% [95% CI, −8.9% to 33.2%]; deep: 18.3% [95% CI, −1.8% to 38.3%]; persistent: 24.4% [95% CI, 7.3%-41.5%]; and combined: 19.7% [95% CI, −0.6% to 40.0%]).

**Figure 3.**
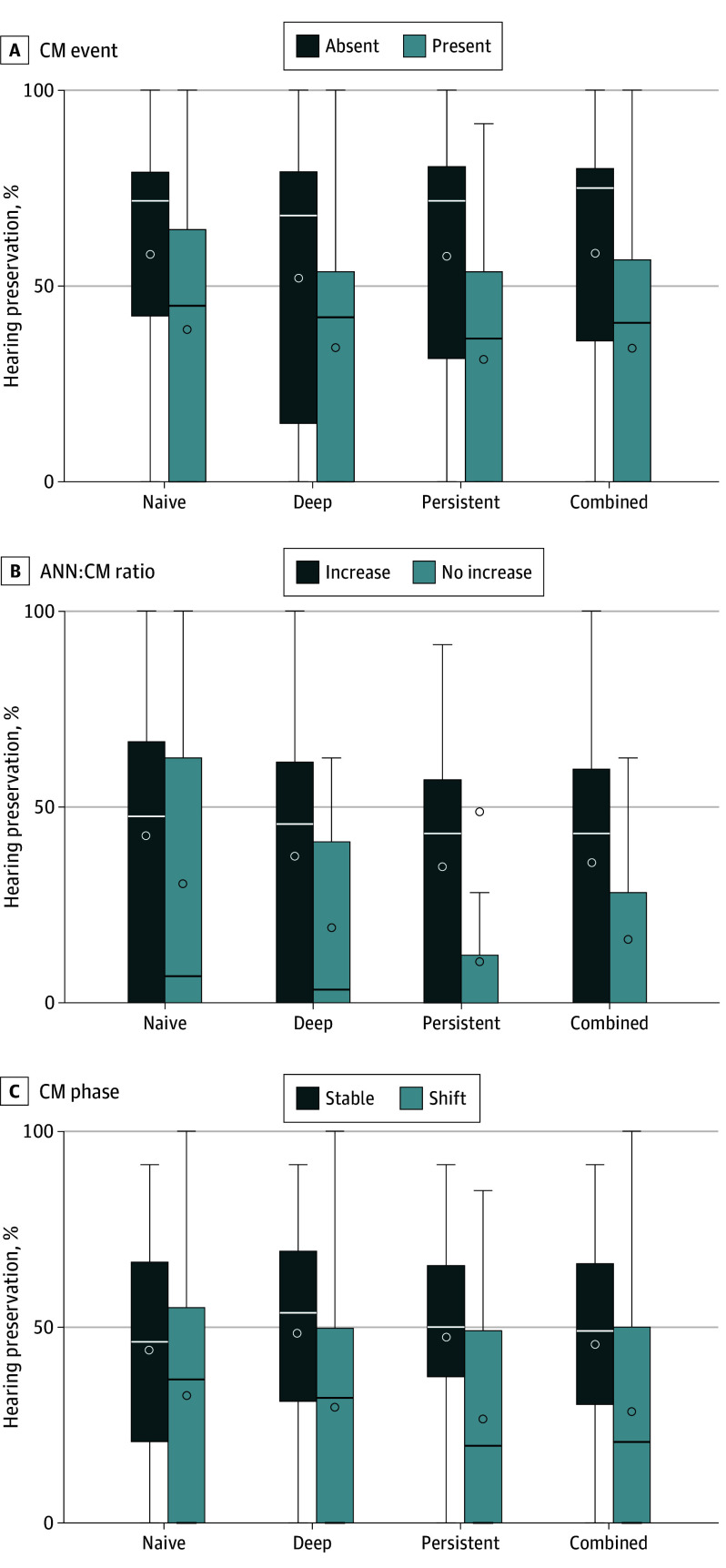
Hearing Preservation Across Different Electrocochleography Features and Classification Paradigms Box-and-whisker diagrams show postoperative hearing preservation, with each column representing a different evaluation paradigm of the cochlear microphonic (CM) events (naive, deep, persistent, and combined). In each row, data are split based on the presence or absence of specific electrocochleography features. Boxes represent the IQRs; whiskers, 1.5 times the IQRs; lines, medians; circles within the boxes, means; and circle outside of the box, outlier. ANN indicates auditory nerve neurophonic.

With regard to CM phase, 65.5% to 82.3% of the events met inclusion criteria, with 65.5% to 70.6% having a stable CM phase. In all tests, events with stable CM phase showed higher hearing preservation compared to those with phase shift (mean difference in HP: naive: 11.6% [95% CI, −5.5% to 28.8%]; deep: 19.1% [95% CI, −0.2% to 38.3%]; persistent: 20.9% [95% CI, 2.9%-38.9%]; and combined: 17.3% [95% CI, −0.3% to 34.8%]). The results of the post hoc analysis are summarized in Figure 3 and Table 2.

**Table 2.** Statistical Summary of the Post Hoc Analysis of Hearing Preservation Across Different Electrocochleography Features and Classification Paradigms

Paradigm	Observations, yes:no (total)^a^	Mean difference (95% CI), %^b^
**CM drop present**		
Naive	88:24 (112)	19.2 (4.4 to 33.9)
Deep	62:50 (112)	18.3 (5.9 to 30.7)
Persistent	62:50 (112)	26.2 (14.3 to 38.0)
Combined	71:41 (112)	24.2 (11.7 to 36.7)
**ANN:CM ratio increase**		
Naive	53:17 (70)	12.2 (−8.9 to 33.2)
Deep	32:10 (42)	18.3 (−1.8 to 38.3)
Persistent	38:8 (46)	24.4 (7.3 to 41.5)
Combined	42:9 (51)	19.7 (−0.6 to 40.0)
**CM phase stable**		
Naive	38:20 (58)	11.6 (−5.5 to 28.8)
Deep	30:15 (45)	19.1 (−0.2 to 38.3)
Persistent	36:15 (51)	20.9 (2.9 to 38.9)
Combined	37:18 (55)	17.3 (−0.3 to 34.8)

Abbreviations: ANN, auditory nerve neurophonic; CM, cochlear microphonic.

^a^ Number of patients per group and in total.

^b^ Mean difference in relative hearing preservation between groups.

## Discussion

Recent research has explored the use of real-time electrocochleography to monitor and improve hearing preservation during cochlear implantation. However, the clinical relevance of electrocochleography remains limited due to the lack of automated software to replace expert interpretation, high signal variability, and the absence of clearly defined surgical interventions.^26,41,42,43^

This international multicenter study addressed these limitations by implementing an automated, objective analysis pipeline for intraoperative electrocochleography signals, aiming to identify possible predictors for individual hearing preservation during cochlear implantation. We statistically evaluated the possible predictive performance of CM events in 112 cochlear implant recipients, identifying key factors associated with hearing preservation. This is crucial for developing clinical software to reliably guide cochlear implantation without requiring expert interaction. This analysis revealed that CM amplitude drops (CM events) are associated with loss of residual hearing when they persisted or when they occurred near the end of insertion. Additionally, patients in which CM events were not accompanied by an increase in ANN:CM ratio, or CM phase shift, tended to show worse hearing preservation outcomes.

Aligning with recent research in the field,^15,16,20,22,24,41^ these findings demonstrate the feasibility of automated electrocochleography analysis for possible intraoperative prediction of individual hearing-preservation outcomes. Moreover, these results highlight the potential of context-aware, simultaneous analysis of multiple electrocochleography features to improve reliability of the approach. As a next step, multimodal prediction models should be developed and evaluated in an interventional study to assess their efficacy to improve hearing preservation by guiding intraoperative decision-making.

### Factors Influencing the Prediction of Hearing Preservation

We used objective, fully automated algorithms for electrocochleography analysis, independent of expert interaction. This enabled statistical evaluation of CM events for association with hearing preservation and systematic testing of different evaluation paradigms (ie, filtering persistence and location).

Logistic regression analysis confirmed CM events as possible predictors for hearing preservation during cochlear implantation. Associations were highest for CM events that were persistent or occurred near the end of electrode insertion. These refined event selection paradigms yielded higher adjusted ORs (between 31.58 and 52.96) compared to naive evaluation of all CM events (adjusted OR, 5.05). Despite large effect estimates, wide upper confidence limits indicated some uncertainty, likely driven by outcome imbalance in the study population and unrecorded variables influencing postoperative hearing loss.^44^ Adjusting for preoperative hearing, age, and implantation center revealed no meaningful outcomes, reinforcing CM events as promising biomarkers for real-time monitoring of cochlear health—especially when compared to surgeons’ self-assessment and tactile perception.^45,46^

The finding that CM events near the end of insertion were associated with loss of residual hearing aligns with temporal bone studies identifying the cochlear region beyond 270° as particularly susceptible to mechanical trauma.^47^ Furthermore, persistent CM events likely reflect irreversible cochlear damage or obstruction of sound transmission.^9,14,41^ Although persistence cannot be evaluated in real time, these findings support intervention protocols, in which insertion is resumed once the amplitude recovers.^17^ Merging both deep and persistent events was associated with slightly improved sensitivity, indicating that strict exclusion rules (eg, completely rejecting basal or transient events) may overlook some cases of loss of residual hearing. Meanwhile, evaluating CM events in the basal-most region of the cochlea proved challenging, possibly due to overall low signal amplitudes rendering minor fluctuations more likely to trigger false detection of events.^18^ Potential strategies to improve the interpretation of basal CM events include parallel recordings at the round window as a reference^48^ or separation of neural and hair cell potentials.^11^

To address these issues (ie, to better differentiate negligible CM events from those indicating loss of residual hearing), emerging evidence proposes the use of additional electrocochleography features.^16,49^ Specifically, an increasing ANN:CM ratio has been consistently associated with preserved neural function (ie, CM events that are irrelevant for the prediction of hearing outcomes).^16,20^ On the other hand, varying observations were made regarding CM phase, and it remained unclear whether a changing or stable phase during CM events is more indicative of hearing loss.^16,22,23^ One interpretation might be that a stable CM phase during an event reflects undisturbed cochlear sound transmission, implying no mechanical distortion of the basilar membrane by the electrode array.^22^ The present results support these hypotheses, as average hearing preservation was consistently higher when CM events were associated with an increasing ANN:CM ratio or stable CM phase. The effect estimates were most pronounced for persistent events, in which the ANN:CM ratio could distinguish different degrees of poor hearing preservation at the group level (mean difference in HP, 24% [95% CI, 7.3%-41.5%]). Results for the CM phase were similar (mean difference in HP, 21% [95% CI, 2.9%-38.9%]). Since average hearing preservation was lower in both cases compared to observations without any CM event, additional features may be useful to distinguish partial from minimal hearing preservation, or between different mechanisms of hearing loss. This distinction could aid in intraoperative decision-making by indicating whether electrode repositioning is needed to resolve impaired sound transmission or whether insertion should be halted to prevent complete hearing loss.

Although first protocols for responding to CM events—such as pausing, retracting, or abandoning further insertion—have shown promise, it remains unclear how the electrode tip actually moves during such manual maneuvers.^15^ With the advent of robotic-assisted insertion, however, electrocochleography-based interventions may be applied more systematically, as robotic systems offer slow, continuous insertion.^50,51^ This controlled environment may enhance both the reliability of electrocochleography signal interpretation and the surgeon’s ability to react to signal changes in a precise and reproducible manner.

### Limitations

Of 145 patients with preoperative hearing thresholds at 500 Hz with at least 85 dB HL, 112 (77%) showed CM responses above 5 μV. Analysis of the ANN:CM ratio and CM phase required additional filtering, further reducing the dataset by 20% to 32% and 18% to 34%, respectively. This underscores the need for complementary measurements such as impedance data when electrocochleography cannot be measured.^44,49^

Methodological limitations include possible artifacts in impedance-based insertion estimates, variability in manual insertions, and potential insertion of the electrode array beyond the tonotopic 500 Hz region, which may cause misleading CM drops (especially under high-intensity stimulation).^52^ A lower-frequency stimulus (eg, 250 Hz) might better target apical regions. In addition, adjusting for preoperative PTA may introduce statistical bias, as it was used to calculate relative hearing preservation, and the multivariable models may be susceptible to overfitting. Finally, residual hearing loss may occur postoperatively and not necessarily intraoperatively.^8,19^

## Conclusions

Hearing preservation during and after cochlear implantation is an emerging yet unresolved clinical priority. This international multicenter cross-sectional study demonstrates the feasibility of automated electrocochleography analysis for possibly predicting individual residual hearing outcomes during cochlear implantation. Persistent CM amplitude drops, or those occurring near the end of electrode insertion, are especially associated with poor hearing preservation. Findings suggest that additional signal features, such as the ANN:CM ratio and CM phase, may enhance the interpretation of cochlear microphonic responses. Interventional studies are needed to evaluate the clinical utility of future iterations of those algorithms in supporting intraoperative decision-making and improving hearing preservation during cochlear implantation.

## Notes

Supplement.
Data Sharing Statement
