# Supplementary material for: Electrocochleography for Monitoring Hearing Preservation During Cochlear Implantation
Source: JAMA Otolaryngol Head Neck Surg. 2025 Nov 20;152(2):117–25. doi: 10.1001/jamaoto.2025.4044 (PMC12635923; doi:10.1001/jamaoto.2025.4044)
Supplement: Supplement. — Data Sharing Statement [file jamaotolaryngolheadnecksurg-e254044-s001.pdf]

Data Sharing Statement

Andonie. Electrocochleography for Monitoring Hearing Preservation During Cochlear Implantation. *JAMA Otolaryngol Head Neck Surg*. Published November 20, 2025. doi:10.1001/jamaoto.2025.4044

Data
Data available: Upon request (due to differing local regulations, part, but not all of the data may be made available)
Data types: Deidentified participant data
How to access data: raphael.andonie@unibe.ch
When available: After a separate data sharing agreement has been reached with each of the involved institutions

Supporting Documents
Documents available: Yes
Document types: Statistical code (Python)
How to access documents: raphael.andonie@unibe.ch
When available: With publication

Additional Information
Who can access the data: Anyone requesting data
Types of analyses: For non-commercial purposes only
Mechanisms of data availability: With investigator support after a separate data sharing agreement has been reached with each of the involved institutions
